# New applications of sustainable, scalable, standardized, and cost-effective human biomaterials for cell-based assays, tissue engineering, and regenerative medicine

**DOI:** 10.3389/fbioe.2025.1676369

**Published:** 2026-01-02

**Authors:** Rebekka J. S. Salzmann, Sandra Domazet, Sonia Prado-López, Seyda Kigili, Winfried Neuhaus, Andreas Brachner, Gerda Egger, Lorenzo Moroni, Johannes Hackethal

**Affiliations:** 1 Maternal and Child Health Department Pediatric Hematology, Oncology and Stem Cell Transplant Centre, University of Padua, Padua, Italy; 2 Istituto di Ricerca Pediatrica “Città della Speranza”, Padua, Italy; 3 Department of Pathology, Medical University of Vienna, Vienna, Austria; 4 Ludwig Boltzmann Institute Applied Diagnostics, Vienna, Austria; 5 Institute of Solid-State Electronics, Faculty of Electrical Engineering and Information Technology, TU Wien, Vienna, Austria; 6 Competence Unit Molecular Diagnostics, Centre for Health and Bioresources, AIT - Austrian Institute of Technology GmbH, Vienna, Austria; 7 Faculty of Medicine and Dentistry, Danube Private University, Krems, Austria; 8 Department of Complex Tissue Regeneration, MERLN Institute for Technology-Inspired Regenerative Medicine, Maastricht University, Maastricht, Netherlands; 9 THT Biomaterials GmbH, Vienna, Austria; 10 Austrian Cluster for Tissue Regeneration, Vienna, Austria

**Keywords:** human placenta, biomaterials, extracellular matrix, colorectal cancer spheroids, cell adhesion, blood brain barrier, 3R

## Abstract

Human-derived biomaterials offer several advantages over animal-derived or synthetic alternatives, including improved biocompatibility, ethical acceptability, sustainability, and clinical translatability. Here we present new applications of human placenta-derived materials – specifically HUMAN PLACENTA substrate, collagen type-I, and Laminin-111 – as 2D coating materials and 3D matrices for the cultivation of spheroids and adherent cells. Collagen type-I coatings supported colorectal cancer spheroid formation without the need for growth-factor supplementation. Lm-111 significantly enhanced NIH3T3 fibroblast adhesion compared with poly-L-lysine and rat-tail collagen type-I, performing comparably to bovine fibronectin. In a transwell blood–brain barrier model, HUMAN PLACENTA substrate coatings enabled confluent endothelial monolayers with transendothelial electrical resistance values not significantly different from the conventional human collagen type-IV/bovine fibronectin mixture. Across these *in vitro* models, placenta-derived materials performed comparably or better than conventional animal-derived and synthetic coatings, supporting robust cell viability, adhesion, and barrier formation. Due to their human origin, these biomaterials exhibit reduced biological complexity while enhancing biocompatibility and translational relevance. Therefore, they provide a sustainable, ethically acceptable alternative for advanced cell culture systems.

## 1 Introduction

Human-derived biomaterials are increasingly preferred over animal-derived or synthetic materials in research and clinical translation due to ethical considerations, closer relevance to human biology, alignment with emerging regulatory frameworks and improved sustainability (Ali et al., 2024; Liu et al., 2021; Malakpour-Permlid et al., 2025). Among human tissues, the placenta represents a particularly attractive, yet under-utilized tissue. It is abundant, typically discarded as medical waste and non-invasively collected after birth, making it a sustainable and ethically acceptable origin of human extracellular matrices (ECM), provided that informed consent and appropriate regulatory standards are in place (Moore et al., 2017; Yoshizawa and Hird, 2020). Placenta-derived ECM components contain structural proteins and bioactive molecules directly relevant to human cell culture and disease modelling. This improves the physiological relevance and translational potential of *in vitro* systems (Biswas et al., 2023; Chao et al., 2022; Roy et al., 2022).

Animal-derived materials such as Matrigel™ or laminin-111 are obtained from small amounts of tumor tissue and rely on animal farming. On the other hand, human stem-cell-derived matrices and decellularized stem-cell cultures can provide Xeno-free scaffolds, but their use is limited by high production costs, donor-to-donor variability, and ethical constraints related to stem-cell sourcing and manipulation (Schäffers et al., 2025; Sugarman et al., 1995). In contrast a single human placenta is naturally available after birth, weighs roughly half a kilogram and can yield multiple biomaterials from a single batch (Passaniti et al., 2022). It provides an ECM naturally adapted to support rapid growth, angiogenesis, and immune tolerance during pregnancy, properties advantageous for regenerative and disease-modelling applications. Human-derived matrices such as those derived from placenta closely replicate native human ECM in both biochemical composition and mechanical behavior. They contain human-specific isoforms of collagens, laminins, fibronectins, and glycosaminoglycans with appropriate cross-linking density and elasticity, supporting integrin-mediated cell adhesion and signaling (Moghassemi et al., 2025; Roy et al., 2022). Moreover, placental ECM composition is relatively stable across donors, reducing batch variability compared with stem-cell–derived products, and its isolation requires no lengthy *in vitro* expansion or differentiation steps, making it scalable and cost-effective (Roy et al., 2022). Animal-derived materials such as bovine fibronectin (bFN), murine collagen and Matrigel™ provide similar structural support but differ in post-translational modifications, glycosylation patterns, and stiffness profiles. These differences can alter receptor binding and trigger xenogeneic immune responses, limiting translational predictability (Li et al., 2021; Wang et al., 2020). Synthetic materials, such as polyethylene glycol or poly-L-lysine (PLL) based coatings, offer mechanical tunability and batch reproducibility but lack inherent bioactivity, requiring chemical functionalization with peptides or growth factors (GFs) to mimic ECM signaling (Lebaudy et al., 2023; Manouchehri et al., 2021). In summary, human-derived biomaterials combine biological fidelity with ethical acceptability, while maintaining scalability and compositional complexity unattainable by purely synthetic scaffolds.

Interest in the ECM and its components continue to grow in tissue engineering and disease modelling due to its role in regulating key cellular processes and its involvement in disease progression (Nieto and Lutolf, 2011). Particularly in cancer it shapes the tumor microenvironment and promotes metastasis (Boudreau and Bissell, 1998; Courbot and Elosegui-Artola, 2025; Escobedo-Lucea et al., 2012). One major application is the generation of three-dimensional (3D) tissue constructs such as spheroids and organoids (Zhao et al., 2022). These 3D models provide a physiologically relevant alternative to traditional two-dimensional (2D) cultures, which fail to reproduce the spatial organization and mechanical properties of native tissues (Rauner et al., 2025). Conventional 3D culture protocols rely on exogenous factors such as epidermal growth factor (EGF), basic fibroblast growth factor (bFGF), and insulin-transferrin-selenium (ITS) which increase the cost and system complexity (Hebert et al., 2009). Animal-derived ECM products such as Matrigel™ from a mouse sarcoma or collagen type-I typically extracted from rat or bovine tendons, are widely used but limited in clinical translation and reproducibility due to their non-human origin (Gjorevski et al., 2016). Synthetic ECM alternatives, though animal-free, often lack the biological activity necessary for effective cell signaling and require extensive chemical modification to approximate native ECM functions (Mukhopadhyay, 2023).

We recently described a roadmap toward the sustainable use of placenta-derived biomaterials and demonstrated first applications in cell culture and clinical translation. To address these challenges, we evaluated placenta-derived COL-I as a coating for CRC spheroid formation as an alternative to ultra-low adherence plates supplemented with GFs. Because surface coatings are critical for cell adhesion, we compared placenta-derived Lm-111 with commonly used animal-derived and synthetic coatings - rat-tail collagen type-I (rCol-I), bFN, and PLL - for fibroblast attachment. Moreover, we explored hpS as a coating substrate in a transwell BBB *in vitro* model using the human cerebral microvascular endothelial cell line hCMEC/D3 compared to a conventional human collagen type-IV (hCol-IV) and bFN mix (Gerhartl et al., 2020). We assessed the barrier function using transendothelial electrical resistance (TEER). These studies aim to address the growing demand for biologically relevant, Xeno-free alternatives that better mimic human physiology *in vitro*. By exploiting the unique structural and biochemical properties of placenta-derived biomaterials, we propose sustainable, human-relevant culture systems that advance cell-based assays, tissue engineering, and regenerative medicine.

## 2 Materials and methods

### 2.1 COL-I coatings for CRC spheroid formation

The two CRC cell lines HT-29 (ATCC, RRID: CVCL_0320) and Caco-2 (#HTB-37, ATCC) were cultured in complete medium composed of DMEM-F12 (Lonza, United States) supplemented with 3.151 g/L glucose, l-glutamine, 10% (v/v) bovine serum EU standard (Lonza, United States) and penicillin/streptomycin (Lonza, United States), at a working concentration of 100 U of potassium penicillin and 100 μg/mL streptomycin sulphate. All cultures were maintained in an incubator containing 5% CO_2_ at 37 °C and media was changed every 48 h.

HT-29 at a passage number of 32 and Caco-2 at a passage number of 17 were seeded at a density of 5,000 cells/well in a total volume of 150 μL of complete medium on either uncoated 96-well plates (#650180, CELLSTAR®) or on 96-well plates coated with 40 μg/mL and 100 μg/mL COL-I (#THT0101, THT Biomaterials). For a positive control of 3D spheroid formation, 10,000 Caco-2 cells/well were seeded on ultra-low attachment 96-well plates (Nunclon™ Sphera™, Thermo Fisher Scientific), supplemented with 20 ng/mL human recombinant epidermal growth factor (EGF, #GF144, Sigma-Aldrich Sigma-Aldrich), 10 ng/mL human recombinant basic fibroblast growth factor (bFGF, #GF003AF, Merck KGaA) and 5 μg/mL human ITS (#41400045, Gibco™).

Cell proliferation and viability assays were performed in four biological replicates using the Cell Counting Kit-8 (CCK-8) assay (MedChemExpress) and the CASY Cell Counter and Analyzer (OLS OMNI Life Science) (Ell et al., 2024; Magar et al., 2021). The CCK-8 reagent was directly added to the cell samples at 10% (v/v), followed by incubation for 4 h at room temperature. Absorbance was measured at 450 nm using a microplate reader (Byonoy GmbH). The assay is based on the reduction of the water-soluble tetrazolium salt WST-8 by mitochondrial dehydrogenases in viable cells to produce an orange formazan dye, the intensity of which is linearly related to cell viability. Cell viability was calculated using standard normalization to blank and control signals. CASY measurements were performed by detaching cells with Accutase (Sigma-Aldrich), centrifuging, resuspending in culture medium, diluting in CASYton buffer, and analyzing via the CASY Excel system. Data was collected after 24 h, 48 h, 72 h, and 96 h in culture. Statistical analysis was conducted using a two-way ANOVA Tukey’s multiple comparisons test with a 95% confidence interval (GraphPad Prism V10).

Spheroid formation was analyzed by acquiring brightfield images at 4x and 10x magnification using a NIKON ECLIPSE TE200 inverted microscope and Hamamatsu camera system and quantified using Fiji (Schindelin et al., 2012) by selecting a defined area and, defining a threshold and a subsequent particle analysis with parameters set to 200–1,200 μm^2^ size and 0.5–1.0 circularity. Output data included spheroid count, area, perimeter, and circularity, and were exported as .csv files. Images from uncoated wells, where no spheroids formed, served as negative controls. Spheroid formation experiments were conducted in technical triplicates with five sections per well being counted.

### 2.2 Lm-111 coatings for NIH3T3 fibroblast cell adhesion

Four different coating substances were compared in eleven biological replicates with three technical replicates in a 96-well plate format. All following steps use a volume of 100 µL per well and the plate is always incubated for the specified time in a humidified incubator at 37 °C, 5% CO_2_.

To coat the plates all wells were filled with the coating substance, incubated and then washed twice with 1x PBS. rCol-I (#C7661, Sigma) was diluted 1:30 in 1x PBS from a 3.34 mg/mL stock concentration to 111.3 μg/mL and incubated for 4 h. PLL (#P1524, Sigma-Aldrich) stock solution (0.1%) was diluted 1:2 in 1x PBS and incubated for one to 4 h. bFN (#341631, Sigma-Aldrich) was diluted 1:200 in 1x PBS and incubated 4 h. Lm-111 (#THT0201, THT Biomaterials) was used at 111.3 μg/mL in 1x PBS and incubated for 4 h.

Per well, 6,000 NIH3T3 fibroblasts at passage number 30 were seeded in medium and incubated for 20 min. Cells that didn’t adhere were removed by inverting the plate and centrifuging it top side down at 50 *x g* for 5 min. Adherent cells were fixed with 100 µL methanol (100%) per well for 10 min at room temperature. Methanol was removed and cells were stained with 0.5% crystal violet solution in 70% ethanol for 20 min. The staining solution was discarded, and the wells were washed with water until the washing water remains clear. The wells were dried, and the staining solution was extracted with 0.1% SDS in water for 10 min on a horizontal shaker. The absorption at OD590 was measured with OD405 for background correction. Statistical analysis was performed using repeated measures one-way ANOVA Tukey’s multiple comparisons test with a 95% confidence interval (GraphPad Prism V10).

### 2.3 Comparison of hpS with hCol-IV/bFN mix as coating substrate for a transwell BBB model

The human immortalized cerebral microvascular endothelial cell line hCMEC/D3 (Weksler et al., 2005) was cultured on 0.5% gelatine-coated culture flasks (#22.151.02; SERVA Electrophoresis GmbH, Heidelberg, Germany) in EBM-2 endothelial basal media (#CC3156; Lonza, Basel, Switzerland) supplemented with 5% FBS, 100 U/mL Penicillin, 100 μg/mL Streptomycin, 10 mM HEPES (#H0887; Sigma-Aldrich), 5 µg/mL ascorbic acid (#A4544; Sigma-Aldrich) and 1 ng/mL human basic fibroblast growth factor (bFGF; #F0291; Sigma-Aldrich). hCMEC/D3 were split weekly at full confluence at a ratio of 1:3. All experiments were performed with Greiner ThinCert® 0.4 µm pore size membrane inserts in 24-well multiwell plate format (#662641; Greiner Bio-One). Prior cell seeding, each ThinCert® was incubated apically with 100 µL of coating solution mix, either containing 400 μg/mL hCol-IV (#C5533; Sigma-Aldrich) and 250 μg/mL bFN (#F1141, Sigma-Aldrich) dissolved in 1x DPBS, or hpS (#THT030110; THT Biomaterials) diluted 1:7 in 1x DPBS, overnight at 37 °C. After incubation with the coating mixtures, inserts were washed twice with sterile 1x DPBS and immediately used for cell seedings. hCMEC/D3 were seeded at a density of 40,000 cells/cm^2^. Medium was changed on days three and six post-seeding. On day six, FBS concentration was reduced to 0.25% (v/v). Transendothelial electrical resistance measurements were performed with an EVOM volt-ohm meter device (World Precision Instruments, Sarasota, FL, United States) and STX2 chopstick electrodes (Merck Millipore). Measurements were done after media exchange and equilibration for 40 min at room temperature. Measurements were performed in three biological-with three technical replicates per condition at passage numbers 30, 26 and 28. TEER values (Ω x cm^2^) were calculated by subtracting the mean resistance value of (coated, cell-free) blank ThinCerts® and multiplication with the membrane surface area of 0.336 cm^2^. Statistical analysis was performed using a student’s t-test using GraphPad prism.

## 3 Results

### 3.1 COL-I coatings support CRC spheroid formation

**FIGURE 1:**
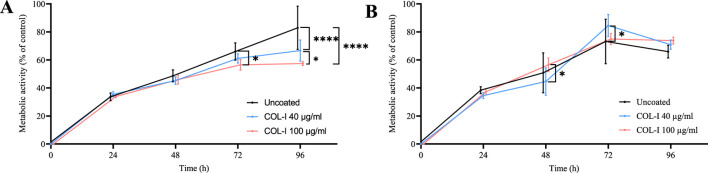
Cell viability measured as metabolic activity with CCK-8 compared to internal control of **(A)** Caco-2 and **(B)** HT-29 cell lines cultured on uncoated plates (black), plates coated with 40 μg/mL (blue) and 100 μg/mL COL-I (red) after 24 h, 48 h, 72 h, and 96 h. Data represent mean ± SD of four biological replicates (n = 4). Statistical analysis was performed using a two-way ANOVA Tukey’s multiple comparisons test with a 95% confidence interval (GraphPad Prism V10). After 72 h, Caco-2 metabolic activity on uncoated plates was significantly higher than on 100 μg/mL COL-I coated plates (p = 0.0157), and after 96 h it was significantly higher than on both 40 μg/mL and 100 μg/mL COL-I coated plates (p < 0.0001). Additionally, metabolic activity on 40 μg/mL COL-I coated plates was significantly higher than on 100 μg/mL COL-I coated plates (p = 0.0191). For HT-29 cells, after 48 h metabolic activity was significantly higher on 100 μg/mL COL-I coated plates compared to 40 μg/mL COL-I (p = 0.0218), and after 72 h activity on 40 μg/mL COL-I coated plates was significantly higher than uncoated plates (p = 0.0302).

As an indicator for the amount of actively proliferating cells the metabolic activity of the CRC cell lines Caco-2 (Figure 1A) and HT-29 2D (Figure 1B) was assessed in 2D monolayer cultures grown on uncoated, 40 μg/mL and 100 μg/mL COL-I coated 96-well plates at 24 h, 48 h, 72 h and 96 h using the CCK-8 assay.

For Caco-2 cells no statistically significant difference in metabolic activity was observed among the three different conditions at 24 h and 48 h. However, after 72 h, cells cultured in 100 μg/mL COL-I coated plates show a significantly lower proliferation compared to the uncoated control (p = 0.0157). At 96 h, both 40 μg/mL and 100 μg/mL COL-I coatings show a significantly lower proliferation compared to the uncoated control (p < 0.0001).

In contrast, HT-29 cells showed a significantly higher metabolic activity at 48 h on 40 μg/mL compared to 100 μg/mL COL-I (p = 0.0218). At 96 h the activity on the 40 μg/mL COL-I coated plate was significantly higher than on the uncoated control (p = 0.0302).

**FIGURE 2:**
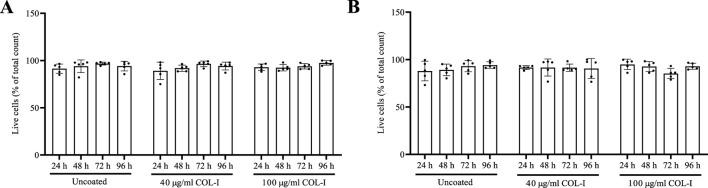
Percentage of live cells of total cell count of **(A)** Caco-2 and **(B)** HT-29 cell lines cultured on uncoated, 40 μg/mL and 100 μg/mL COL-I coated plates after 24 h, 48 h, 72 h and 96 h. Data represent mean ± SD of four biological replicates (n = 4). Statistical analysis was performed using a 2-way ANOVA Tukey’s multiple comparisons test with a 95% confidence interval (GraphPad Prism V10). No significant differences were found.

The percentage of live cells in both cell lines across all treatments and time points was measured using CASY (Figure 2), and no significant differences were observed.

**FIGURE 3:**
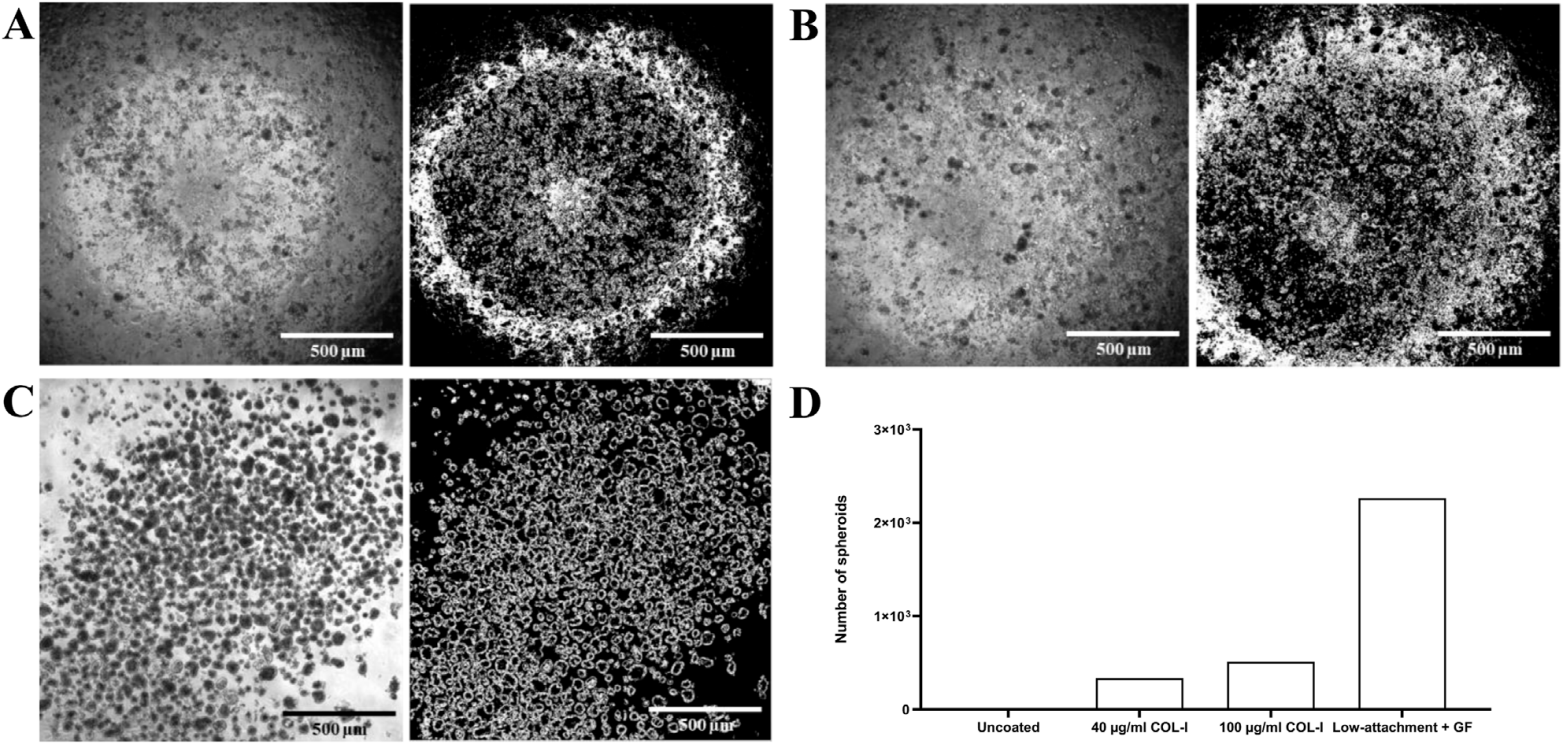
Caco-2 spheroid formation in 40 μg/mL and 100 μg/mL COL-I coated plates and ultra-low attachment plates supplemented with growth factors (GFs) after 24 h in culture. Brightfield images at ×4 magnification of Caco-2 spheroids in **(A)** 40 μg/mL COL-I, **(B)** 100 μg/mL COL-I and **(C)** ultra-low attachment plates supplemented with GFs. **(D)** Number of spheroids formed per 23.312 mm^2^ in uncoated plastic, 40 μg/mL and 100 μg/mL COL-I and ultra-low attachment plates supplemented with GFs. Spheroids were counted using Fiji (ImageJ) by applying size (200–1,200 μm^2^) and circularity (0.5–1.0) filters to identify rounded structures. Data represents one biological replicate (n = 1).

After 24 h in culture, spheroid formation was observed on COL-I coated plates at both 40 μg/mL and 100 μg/mL, as well as on ultra-low attachment plates supplemented with growth factors (EGF, bFGF) and ITS, which served as a positive control (Figures 3A–C). Caco-2 cells did not form spheroids on uncoated 96-well plates without supplements. The 40 μg/mL COL-I coating produced 335 spheroids per 23.312 mm^2^, while the 100 μg/mL COL-I coating generated 510 spheroids per 23.312 mm^2^ which corresponds to 1.5 times more than the 40 μg/mL coating (Figure 3D). In the positive control, 2,263 spheroids per 23.312 mm^2^ were formed, representing 4.43 times more than on the 100 μg/mL COL-I coating and 6.75 times more than the on 40 μg/mL COL-I coating.

**FIGURE 4:**
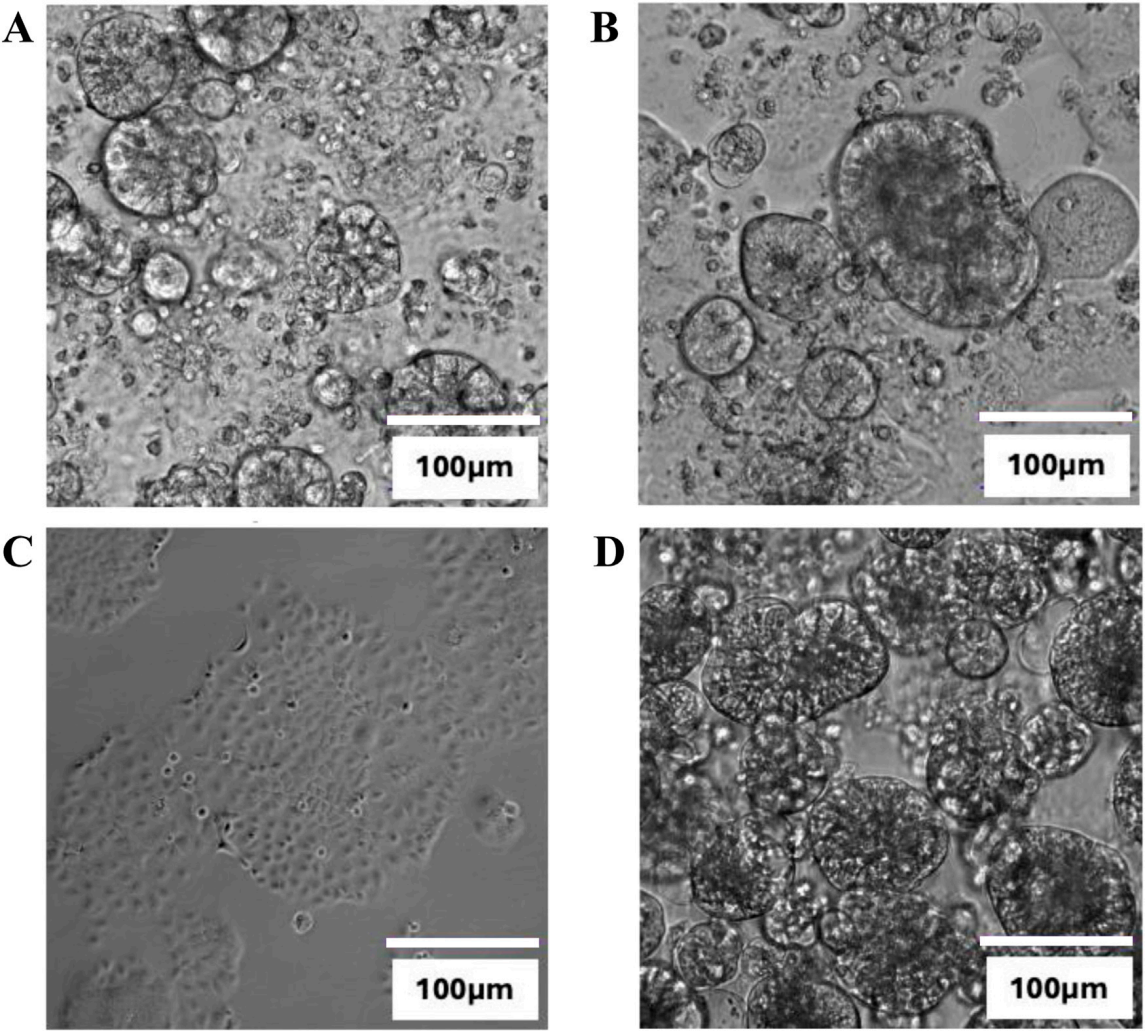
Brightfield microscopy images at ×40 magnification of Caco-2 cells in culture after 24 h on 96-well plates coated with **(A)** 40 μg/mL COL-I, **(B)** 100 μg/mL COL-I **(C)** uncoated and **(D)** and ultra-low attachment plates supplemented with growth factors (GFs) as a positive control for spheroid formation. Data represents one biological replicate (n = 1).

Spheroid formation of Caco-2 could be observed in the plates that were coated with 40 μg/mL and 100 μg/mL COL-I (Figures 4A,B) without the addition of GFs. The higher concentration of COL-I coating seems to be of advantage for spheroid formation. In the negative control on uncoated plates (Figure 4C) no spheroid formation was observed and in the positive control condition on the ultra-low attachment plates supplemented with GFs spheroids did form (Figure 4D).

### 3.2 Lm-111 coating supports NIH3T3 fibroblast cell adhesion

**FIGURE 5:**
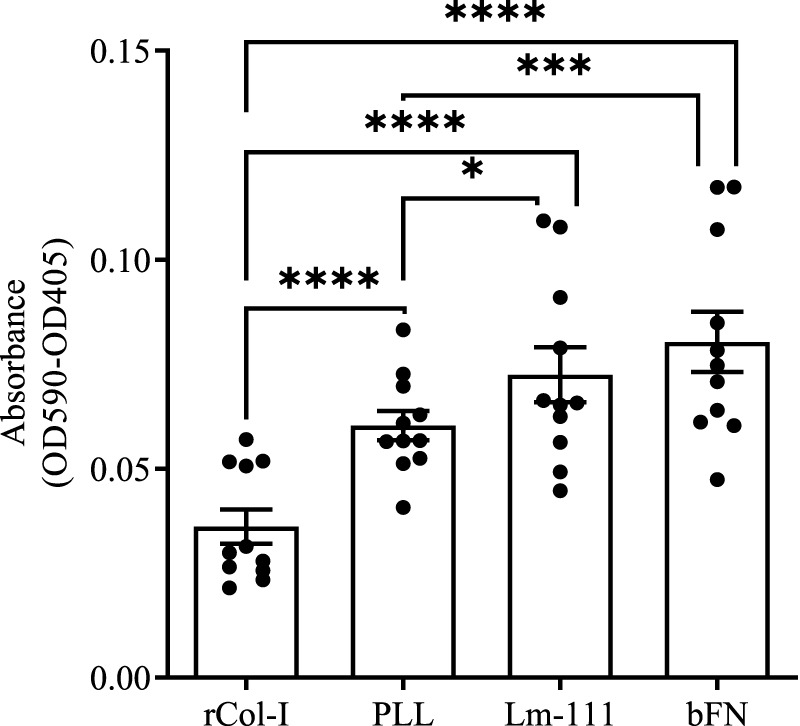
Absorbance OD590-OD405 as measure of amount of attached NIH3T3 fibroblasts on rat-tail collagen type-I (rCol-I), Poly-L Lysine (PLL), human-placenta-derived Laminin-111 (Lm-111) or bovine fibronectin (bFN) coated plates. Statistical analysis was performed using repeated measures one-way ANOVA Tukey’s multiple comparisons test with a 95% confidence interval (GraphPad Prism V10). rCol-I (0.036 ± 0.014) is significantly (****p < 0.0001) lower than PLL (0.060 ± 0.012), Lm-111 (0.072 ± 0.022), and bFN (0.080 ± 0.024). PLL is significantly lower than Lm-111 (*p = 0.0319) and bFN (*p = 0.0002). Lm-111 and bFN do not differ significantly. Data represent mean ± SD of eleven biological replicates (n = 11).

To evaluate the binding affinity of different substrates, NIH3T3 fibroblast were stained with 0.5% crystal violet solution and absorbance values OD490–OD405 were measured for rCol-I, PLL, Lm-111, and bFN. rCol-I exhibited the lowest absorbance (0.036 ± 0.014), which was significantly lower than that of PLL (0.060 ± 0.012, ****p < 0.0001), Lm-111 (0.072 ± 0.022, ****p < 0.0001), and bFN (0.080 ± 0.024, ****p < 0.0001). PLL showed a significantly lower absorbance compared to Lm-111 (*p = 0.0319), and bFN (***p = 0.0002). Lm-111 and bFN exhibited the highest absorbance levels, with no significant difference between them (Figure 5).

### 3.3 hpS coating as a suitable coating alternative for a transwell BBB model

The human cerebral microvascular endothelial hCMEC/D3 cells were differentiated on a transwell membrane that was pre-coated hpS or with hCol-IV/bFN mix.

**FIGURE 6:**
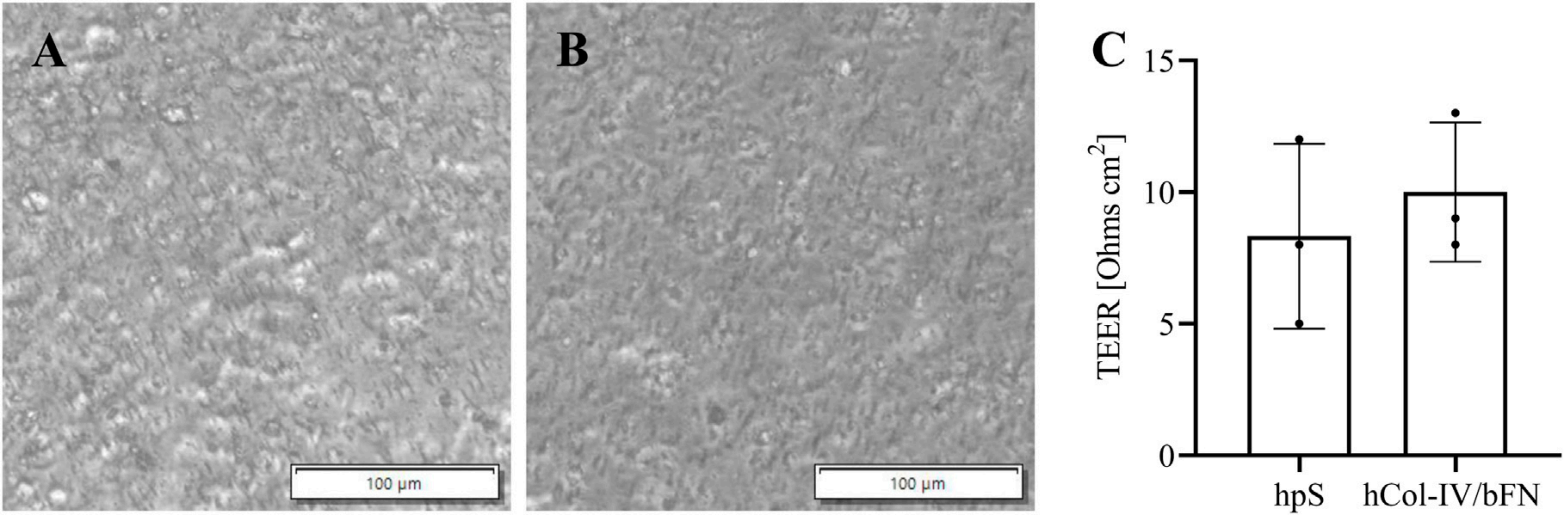
Comparison of substrate coatings for hCMEC/D3 endothelial cell attachment in a BBB model. Bright-field images of cells cultured on transwell membranes pre-coated with **(A)** HUMAN PLACENTA substrate (hpS) or **(B)** a human collagen type-IV and bovine fibronectin mix (hCol-IV/bFN). **(C)** Transendothelial electrical resistance (TEER) measurements assessing barrier integrity. Statistical analysis was performed using Student’s t-test (GraphPad Prism v10), showing no significant differences between groups. Data represent mean ± SD of three biological replicates (n = 3).

Cells grown on the hCol-IV/bFN mix formed a homogeneous, confluent monolayer with a characteristic cobblestone-like morphology typical of mature endothelial cells. The surface appeared smooth and continuous, with no visible gaps or cell-free areas, indicating robust adhesion and uniform cell spreading. These morphological features suggest tight intercellular contacts and a well-established barrier phenotype (Figure 6A).

In contrast, cells cultured on hpS coated membranes also reached confluence but displayed a less uniform appearance. The monolayer showed slightly irregular cell shapes and variable texture, with regions that appeared less compact. This indicates that the hpS coating supports cell attachment and growth but results in slightly less stable or less mature intercellular junctions compared to the hCol-IV/bFN mix coating substrate (Figure 6B).

A subsequent TEER measurement revealed a slightly reduced barrier tightness in the hpS condition; however, the difference between the two coatings was not statistically significant (Figure 6C).

## 4 Discussion

The current findings indicate that human placenta-derived biomaterials can serve as biologically relevant, sustainable alternatives to conventional animal-derived ECMs in advanced cell culture systems (Table 1). Interestingly, the effects of COL-I on the number of metabolically active cells were cell line-dependent. In 2D Caco-2 cultures, 100 μg/mL COL-I reduced proliferation relative to 40 μg/mL and uncoated controls. This decrease may result from increased matrix stiffness at higher COL-I concentrations, which can physically constrain cell spreading and proliferation (Mai et al., 2024). In contrast, HT-29 cells maintained proliferation across both concentrations and exceeded uncoated controls, potentially reflecting differential ECM remodeling capabilities, such as distinct matrix metalloproteinase expression, which modulates proliferation through dynamic ECM turnover (Freitas-Rodríguez et al., 2017). These cell-specific responses may also relate to intrinsic growth kinetics, with HT-29 cells displaying a shorter doubling time of 1 day (Forgue-Lafitte et al., 1989) compared to 2–3 days of Caco-2 cells (Bazzocco et al., 2015). Moreover, COL-I supported rapid spheroid formation within 24 h at both concentrations without requiring specialized plates or GFs. This suggests that human-derived COL-I can facilitate 3D CRC model generation, providing a cost-effective and biologically relevant alternative to both animal-derived ECMs and synthetic matrices. The influence of ECM stiffness and integrin-mediated signaling in modulating proliferation and spheroid formation remains a promising area for further mechanistic investigation. Differential integrin expression between Caco-2 and HT-29 cells, for example, could explain varied responses to COL-I concentration by altering downstream signaling pathways that regulate cell cycle progression.

**TABLE 1:** Comparison of human placenta-derived collagen type-I (COL-I), Laminin-111 (Lm-111) and HUMAN PLACENTA substrate (hpS) and animal rat-tail collagen type-I (rCol-I), human collagen type-IV (hCol-IV), bovine fibronectin (bFN) or synthetic materials like poly L-lysine (PLL) *in vitro*.

Human placenta-derived biomaterials in advanced cell culture systems	COL-I	Lm-111	hpS
Assay	Plate coating for spheroid formation	Plate coating for fibroblast adherence	Coating for a transwell BBB model
Comparison	Ultra-low attachment plates supplemented with GFs (EGF, bFGF) and ITS	rCol-I, PLL, bFN	hCol-IV and bFN mix
Performance	Robust spheroid formation, optimal concentrations are cell line specific	Outperforming rCol-I and PLL; comparable to bFN	Comparable to hCol-IV and bFN mix
Advantage	Reduces costs and complexity introduced by GFs	Xeno-free, 3R, increased biocompatibility and translatability	Xeno-free, 3R, increased biocompatibility and translatability

We have previously shown that Lm-111 enhances the proliferation, viability, and adhesion of Schwann cells (Schuh et al., 2016). In this study we extend these results to evaluate the potential of Lm-111 to support NIH3T3 fibroblast attachment on 96-well plates in comparison with animal-derived and synthetic materials. Among the tested coatings, animal-derived bFN and human Lm-111 outperformed both the synthetic PLL and the animal-derived rCol-I. Due to its human origin, Lm-111 not only supports good cell attachment, but it also represents a biologically active, biocompatible, and ethically favorable substrate with higher clinical translatability. The better performance of Lm-111 and bFN can be explained by their natural bioactivity and cell binding domains. Lm-111 contains multiple domains that bind with integrins (like α1β1 and α6β1) and other adhesion molecules, which supports the formation of strong focal adhesions, cytoskeletal organization, and activation of adhesion and migration signaling (Arimori et al., 2021; Mendonça et al., 2024). bFN is characterized by an RGD motif, which interacts with integrins like α5β1 and αvβ3 and support adhesion (Dalton and Lemmon, 2021). In contrast, PLL is a synthetic polymer that promotes attachment through electrostatic interactions with the negatively charged cell membrane, without interaction with specific receptors (Lebaudy et al., 2023; Manouchehri et al., 2021). Even though rCol-I is an ECM protein it offers fewer accessible adhesion sites for fibroblasts in this setup (Nashchekina et al., 2022). Altogether, the higher attachment on Lm-111 and bFN likely comes from their combination of receptor-specific signaling and mechanical support of adhesion, which is missing in PLL and rCol-I. Future studies should directly compare human recombinant Lm-111, animal-derived Lm-111, and placenta-derived Lm-111, not only in terms of adhesion but also cellular activation and signaling, as these differences may influence differentiation, proliferation, and disease modelling outcomes. Additionally, comparisons with fully defined synthetic matrices, such as peptide-functionalized hydrogels or recombinant laminins, will be needed to understand the advantages of human placenta-derived materials relative to alternatives in terms of bioactivity, reproducibility, and scalability.

In endothelial cultures, cells grown on membranes coated with hpS reached similar confluency and barrier tightness as observed with a hCol-IV/bFN mix coating. This suggests that hpS provides a suitable microenvironment for endothelial attachment, survival, and differentiation. The slightly lower but not significantly different TEER values in hpS might be related to differences in the composition and physical properties of the coatings. The ratio or post-translational modifications of the hpS components such as laminins, collagen or glycoproteins might result in different integrin-signaling or cytoskeletal tension. Moreover, the surface topography or stiffness of the hpS coating might differ slightly from the hCol-IV/bFN mix. These factors might influence tight junctions, morphology, and permeability. Despite these minor differences the comparable TEER-results indicate that hpS supports the formation of a Xeno-free, human-relevant, functional endothelial barrier with a higher translatability. However, while TEER is the most often used parameter to assess barrier integrity, TEER data alone does not sufficiently characterize functional BBB properties (Bolden et al., 2023). Future studies should include additional experiments, such as permeability assays (e.g., FITC-dextran, sodium fluorescein) and expression analyses of tight junction proteins (such as claudin-5 and ZO-1), expression of BBB marker genes, and activity of ABC transporters, to fully validate the functional equivalence of placenta-derived versus animal-derived coatings.

While placenta-derived biomaterials show strong potential, it is important to consider how they compare to other human ECM alternatives such as recombinant and decellularized tissue-derived matrices. Recombinant ECMs, including human recombinant laminins or collagen isoforms, offer excellent batch-to-batch consistency and defined composition but remain cost-intensive and limited in biochemical complexity, as they typically contain only one or a few structural proteins (Naba, 2024). Decellularized human tissues, on the other hand, more closely mimic the native ECM architecture and molecular diversity, but their production is labor-intensive, subject to donor variability, and challenging to scale for large-volume applications (Moura et al., 2022). In contrast, placenta-derived materials have several advantages as they are readily available as medical waste, ethically relatively acceptable, and relatively low-cost to process, while maintaining a rich array of ECM components, GFs, and glycoproteins that support diverse cell functions (Protzman et al., 2023).

Collectively, these findings highlight that human placenta-derived ECM components are promising sustainable and ethical alternatives to Xeno-free materials. Their cell type-specific effects, ability to support 3D culture, and potential to replace synthetic or animal-derived matrices underscore their relevance in advanced *in vitro* modelling. For instance, to test the suitability of hpS-based matrices for 3D PDO cultivation in a proof-of-concept study, PDOs derived from metastatic colorectal cancer were cultivated in hpS-based collagen and fibrin gels compared to standard Matrigel® conditions (Supplementary Figure S1). While cells grown in fibrin/hpS matrices showed reduced organoid forming capacities during a 1-week cultivation period (Supplementary Figure S1A), organoids grown in collagen/hpS showed enhanced growth properties (Supplementary Figure S1B). However, mechanistic studies dissecting integrin signaling, ECM stiffness, and matrix remodeling are needed to optimize their application across diverse cell types and functional assays.

## Data Availability

The original contributions presented in the study are included in the article/Supplementary Material, further inquiries can be directed to the corresponding author.
