# Supplementary material for: New applications of sustainable, scalable, standardized, and cost-effective human biomaterials for cell-based assays, tissue engineering, and regenerative medicine
Source: Front Bioeng Biotechnol. 2026 Jan 2;13:1676369. doi: 10.3389/fbioe.2025.1676369 (PMC12808481; doi:10.3389/fbioe.2025.1676369)
Supplement: Supplementary file 1 [file Supplementaryfile1.docx]

**Materials and Methods**
Patient-derived organoids from a colorectal liver metastasis were generated as described and cultured in standard Matrigel® or in hpS-based matrices combined with fibrin or hCol-I. Cultures were maintained for up to 10 days. Organoid morphology and proliferation were analysed by bright-field microscopy, haematoxylin/eosin staining and Ki67 immunohistochemistry.

**Results**
**Collagen/hpS 3D organoids**

In order to test the suitability of hpS-based matrices for 3D PDO cultivation in a proof-of-concept study, PDOs derived from metastatic colorectal cancer were cultivated in hpS-based collagen and fibrin gels compared to standard Matrigel® conditions (Supplementary Figure 1). While cells grown in fibrin/hpS matrices showed reduced organoid forming capacities during a one-week cultivation period (Supplementary Figure 1A), organoids grown in collagen/hpS showed enhanced growth properties (Supplementary Figure 1B).

Due to the better growth of PDOs in collagen/hpS, we next analysed their histomorphological phenotypes and proliferation rates in comparison to conventional Matrigel® cultures. Thus, FFPE embedded PDOs were stained for HE and the proliferation marker Ki67 (Supplementary Figure 2). Histomorphological analysis revealed similar growth patterns with organisation into gland-like structures (Supplementary Figure 2A). Interestingly, collagen/hpS grown PDOs showed more uniform sphere-like structures with dense cellularity, while Matrigel® PDOs appeared more heterogeneous. Proliferation rates were similarly high in both culture conditions as indicated by Ki67 immunohistochemistry. One-week cultivation of PDOs showed strong morphological differences between Matrigel® and collagen/hpS cultures; while Matrigel® PDOs displayed cloud-shaped, irregular spheres, collagen/hpS PDOs appeared in circular spheres or expanded structures (Supplementary Figure 2B). Notably, cultivation of PDOs in collagen/hpS resulted in significantly larger structures with maximal sizes of 198 µm for Matrigel® PDOs versus 588 µm for collagen/hpS PDOs. In summary, human placenta-derived factors are suitable for organoid cultivation in collagen matrices and allow for long-term cultivation.

**Conclusion**
These results demonstrate the feasibility of hpS-based human-derived matrices as an alternative to Matrigel® for colorectal PDO cultivation. Further optimization and molecular characterization are needed to assess their suitability for high-throughput drug screening and long-term applications.

**
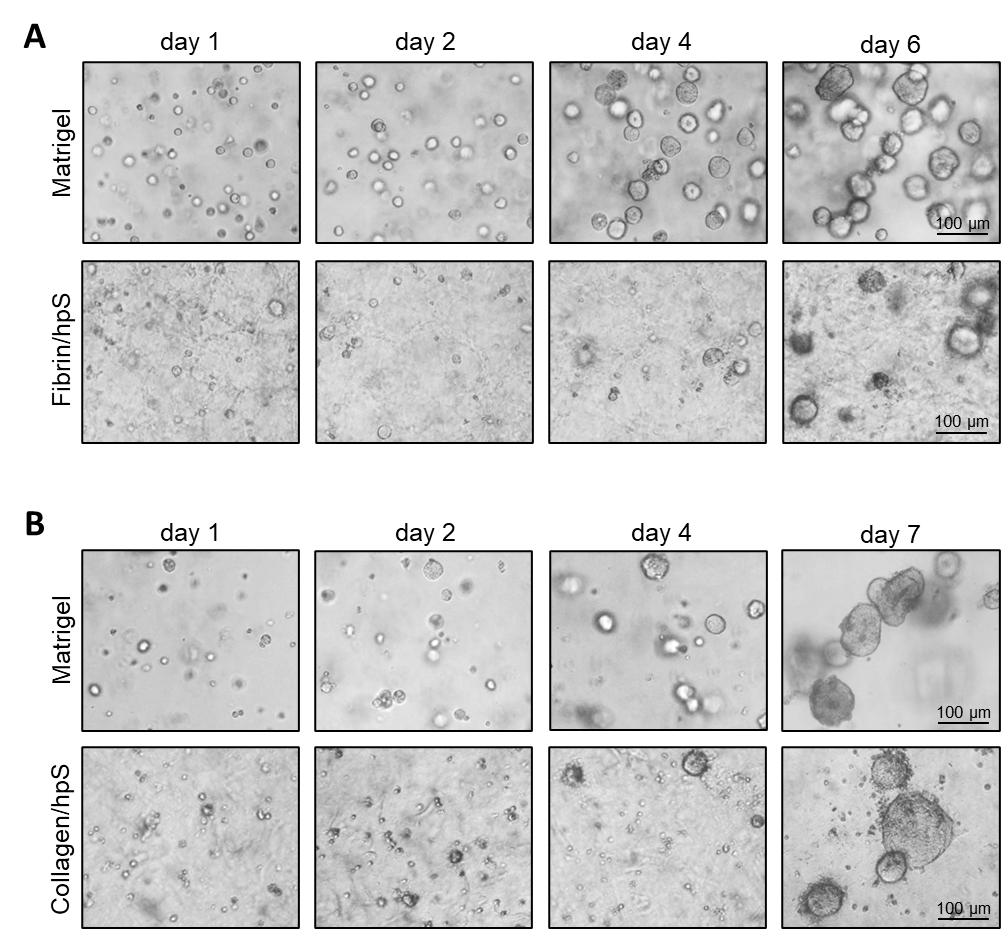
**

**Supplementary figure 1: Organoid cultures in different gels compared to Matrigel. Organoids isolated from a liver metastasis of colorectal cancer were cultured in fibrin/ hpS (A), collagen hpS (B), or Matrigel® for indicated times.**


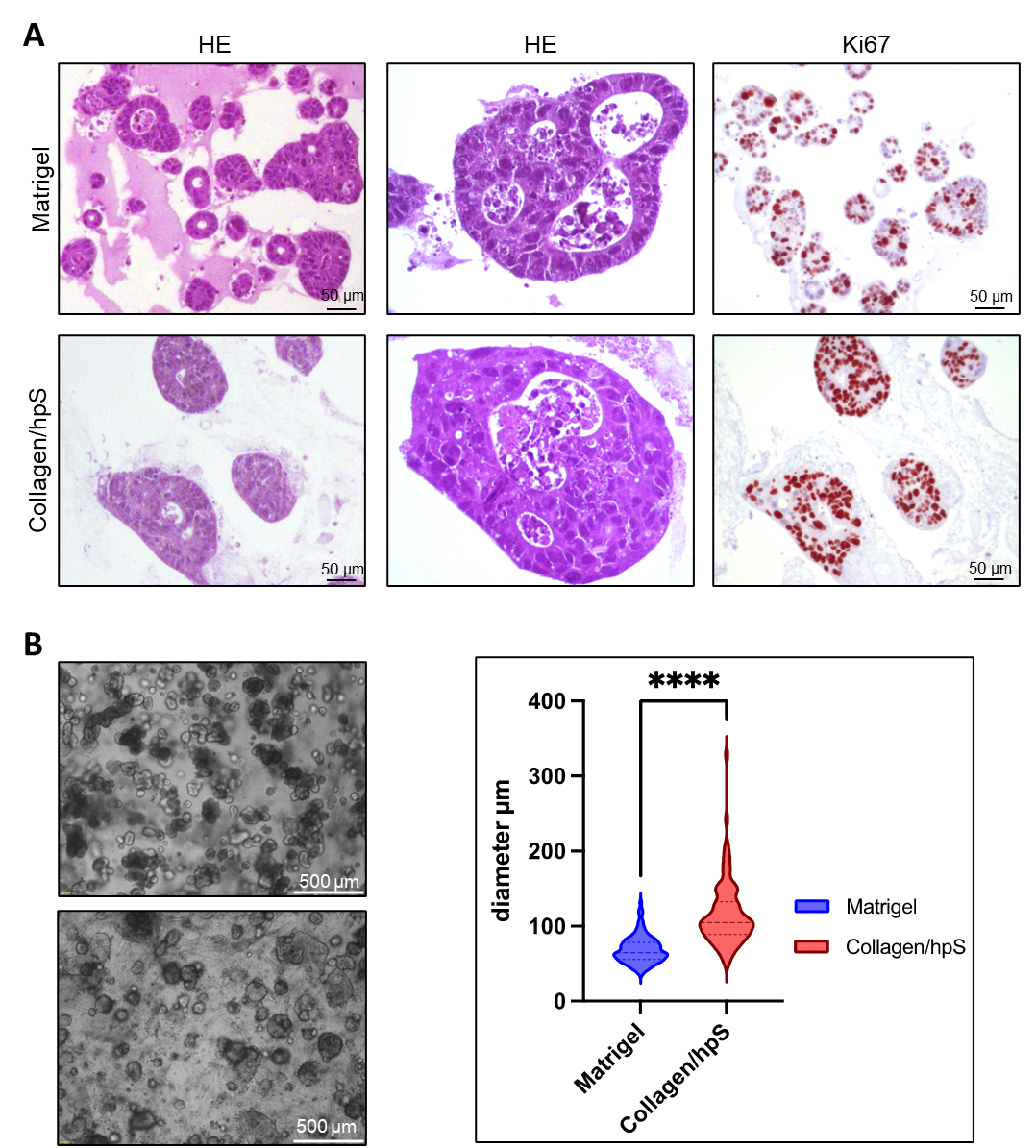


**Supplementary figure 2: Histomorphology of tumor organoids.­­­ (A) Organoids from the indicated culture conditions were fixed in formalin and embedded in paraffin. Staining was done with hematoxilin and eosin (HE, left, middle) or with immunohistochemical staining against Ki67 (right). (B) Bright field microscopic images of PDOs grown for 7 days in Matrigel® (left) or collagen/hpS (middle). The graph on the right shows PDO diameters of individual cultures, measured using Fiji (ImageJ 1.54p) software. Approximately 100 individual PDOs were measured for each condition. Statistics show unpaired T test with Welch’s correction using GraphPad Prism version 10.6.1. **** p <0.0001, replicates (n =3).**

Ki67

H&E

Matrigel™

rCol-I/hpS

Fibrin/hpS


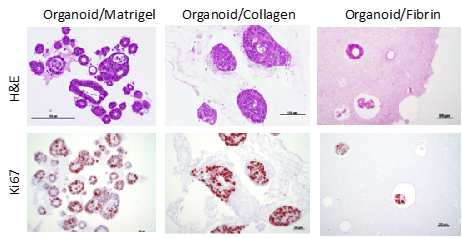


**Supplementary figure 3: Histomorphology of tumour organoids. Organoids from the indicated culture conditions were fixed in formalin and embedded in paraffin. Staining was done with haematoxylin and eosin (H&E) or with immunohistochemical staining against Ki67. Magnification (20x).**
